# Quadruple Therapy Offers High SVR Rates in Patients with HCV Genotype 4 with Previous Treatment Failure

**DOI:** 10.1155/2020/9075905

**Published:** 2020-07-24

**Authors:** Yousry Esam-Eldin Abo-amer, Rehab Badawi, Mohamed El-Abgeegy, Heba Fadl Elsergany, Ahmed Abdelhaleem Mohamed, Sahar Mohamed Mostafa, Hatem Samir Alegaily, Shaimaa Soliman, Sally Elnawasany, Sherief Abd-Elsalam

**Affiliations:** ^1^Hepatology, Gastroenterology, and Infectious Diseases Department, Mahala Hepatology Teaching Hospital, Gharbia, Egypt; ^2^Tropical Medicine Department, Tanta University, Tanta, Egypt; ^3^Hepatology and Liver Transplantation Departments, National Hepatology and Tropical Medicine Research Institute, Cairo, Egypt; ^4^Hepatology, Gastroenterology, and Infectious Diseases Department, Benha Faculty of Medicine, Benha, Egypt; ^5^Department of Public Health and Community Medicine, Menofia University, Menofia, Egypt

## Abstract

**Background and Aims:**

Direct-acting antivirals (DAAs) have made a revolution in hepatitis C virus (HCV) treatment with promising reduction of HCV infection and disease morbidities. However, unfortunately, treatment failure still occurs in about 5–15% of patients treated with DAA‐based combination regimens. The primary aim of the study was to assess the efficacy and safety of a quadruple regimen of (sofosbuvir, daclatasvir, and simeprevir with a weight-based ribavirin) in chronic HCV DAAs-experienced patients.

**Methods:**

This observational, open-label prospective study was carried out on 103 genotype 4 hepatitis C virus-infected patients who failed to achieve SVR12 after sofosbuvir-daclatasvir with or without ribavirin. Patients were treated for three months with sofosbuvir (400 mg), daclatasvir (60 mg), and simeprevir (150 mg) with a weight-based ribavirin dosage (1000–1200 mg/d). Response to treatment was determined by quantitative PCR for HCV at 3 months after the end of treatment (SVR12), and adverse events during the treatment were recorded.

**Results:**

SVR was achieved in 100 patients (97.1%) at week 12 after treatment. No dangerous or life-threatening adverse events were recorded.

**Conclusions:**

Retreatment of HCV genotype 4 patients with quadruple therapy is a good therapeutic option and achieves high response rates with minimal side effects.

## 1. Introduction

Chronic Hepatitis C infection (HCV) constitutes a worldwide health problem. In 2015, the world health organization (WHO) reported 71 million HCV chronically infected people and 1.34 million of chronic liver disease and primary liver cancer-related deaths [1, 2].

Direct-acting antivirals (DAAs) have made a revolution in HCV treatment with promising reduction of HCV infection and disease morbidities [3–8] International liver societies proved DAA-based combination regimens as a new standard of care treatment for chronic HCV [9, 10]. Treatment with at least two DAAs for hepatitis C virus (HCV) genotype 1 or 4 is associated with more than 90% sustained virological response (SVR) rates [11–15].

Chronic hepatitis C patients were treated in 2016 with a combination of 1 to 3 DAAs of 4 groups, with or without ribavirin [16, 17]. It is noteworthy mentioning that NS5B inhibitors have a high resistance barrier because the variables you choose modestly reduce the susceptibility to these drugs and lower fitness. Hence, penetration or relapse is the exception when these medications are given as a monotherapy. On the other hand, NS5A inhibitors and protease inhibitors NS3-4A have low barriers to resistance. When given as monotherapy, they quickly select the appropriate resistance variants. Second-generation NS3-4A and NS5A inhibitors have increased barriers to resistance. They are substantially more active against many but not all variants resistant to first-generation compounds [18].

Unfortunately, treatment failure still occurs in about 5–15% of patients treated with DAA‐based combination regimens. Treatment failure of HCV may be the result of viral factors (HCV genotype and the presence of resistant variants), host factors (fibrosis and portal hypertension), and treatment-related factors (patient's adherence, duration of therapy metabolism of the drugs, and ribavirin use) [19–22].

These viral factors that lead to failure are attributed to the selection existence of HCV viral variants that resist the used DAA [23–26].

Retreatment after DAA failure is a challenge, especially in those for whom NS5A inhibitors-based regimens with cross‐resistance across all members of the drug class [25–27]. Together with the wide spread use of NS5A inhibitors-based regimens, this leads to long-term persistence of resistant variants that convey viral resistance up to 96 weeks after treatment failure [23, 28, 29].

The current updates of the international HCV treatment guidelines recommend a single-tablet combination of SOF plus velpatasvir and voxilaprevir (SOF/VEL/VOX) for 12 weeks as the standard treatment after failure of NS5A-based regimens. Addition of RBV or extension of the treatment duration of the SOF/VEL/VOX regimen, as well as combining SOF with glecaprevir and pibrentasvir (GLE/PIB), could be considered in difficult-to-treat patients [10].

On the other hand, NS3/4A protease inhibitors induce a shorter term of resistance [30], and when added to a high-resistance barrier of sofosbuvir, as well as, to protease inhibitors (that lack cross‐resistance between NS5A inhibitors), they present a reasonable option for retreatment of NS5A‐containing regimen failures [22, 26–34].

It is worth mentioning that due to the limited availability of some newer DAAs in Egypt, the national committee for viral hepatitis management recommended treatment of hepatitis C patients for whom previous SOF/DCV-based regimens have failed with a combination of either SOF plus ritonavir boosted with paritaprevir and ombitasvir (OBV/PTV/r) ± RBV or SOF plus simeprevir (SMV) and DCV ± RBV for 12 or 24 weeks according to RBV eligibility [19].

We aimed in this study to evaluate the efficacy and safety of combining sofosbuvir, daclatasvir, and simeprevir with ribavirin in chronic HCV DAAs-experienced patients.

## 2. Methods

This observational, open-label prospective study was carried out on 103 genotype 4 hepatitis C virus-infected patients who failed to achieve SVR12 after sofosbuvir and daclatasvir with or without ribavirin.

All patients were attending to an insurance hospital clinic and outpatient clinics of a major University hospital and a major research institute during the period of April 2018 to October 2018. All patients had positive quantitive PCR for HCV infection after failure of sustained virological response at 12 weeks after the end of treatment. Institutional ethical committee approval was obtained before the start of the study, and a written consent was taken from every participant. The study protocol conforms to the ethical guidelines of the 1975 Declaration of Helsinki as reflected in a prior approval by the institution's human research committee.

The study enrolled all the patients who agreed to participate and who were within the inclusion criteria of the study. Inclusion criteria were patients aged 18 years or older who are treatment-experienced compensated Child-Pugh A cirrhotic patients and were HCV positive RNA by PCR at 12 weeks after the end of treatment with sofosbuvir and daclatasvir with or without ribavirin.

The exclusion criteria were (a) decompensated cirrhosis, (b) organ transplantation, (c) severe uncontrolled morbidity, e.g., cardiac, (e) malignant Tumors, (f) HIV or hepatitis B virus coinfection, (g) pregnancy or lactation, and (h) receiving contraindicated concomitant drugs for sofosbuvir, daclatasvir, and simeprevir plus ribavirin.

All patients enrolled in this study were evaluated by the complete blood count (CBC), liver function tests, fasting blood sugar (FBS), serum creatinine, HBs-Ag testing, and *α*-fetoprotein (AFP), as well as abdominal ultrasonography.

Patients were treated for 12 weeks with a combination of sofosbuvir (SOF) (400 mg), daclatasvir (DAC) (60 mg), and simeprevir (SIM) (150 mg) with a weight-based ribavirin dosage RBV (1000–1200 mg/d).

Follow-up of the patients was routinely conducted every 4 weeks when the patients attended the clinic to receive their prescriptions. Any side effects noticed or complained by the patients were recorded. If there were side effects, additional visits were recommended.

Complete blood count, liver and kidney function tests, and PCR for HCV were performed before the start of therapy and at weeks 4, 8, and 12 during antiviral treatment.

End of treatment response was determined by quantitative PCR for HCV at end of treatment for each of the study groups.

The primary outcome measures were the number of patients with successful virus eradication at 12 weeks after discontinuation of therapy (SVR12). SVR was defined as negative HCV-RNA 12 weeks after completion of DAA therapy.

The secondary end point was to evaluate the side effects and the safety of this regimen in our patient groups.

### 2.1. Statistical Analysis

Data were expressed in Number (No), percentage (%), mean (x̅), and standard deviation (SD). Kolmogorov–Smirnov and Shapiro–Wilk tests were used to test the normality of different variables. Repeated measures ANOVA (with the Bonferoni correction) with the Mauchly test as a sphereicty test were used for comparison among three or more consecutive measures in the same group of quantitative variables. Assumed spherecity was used for normally distributed data, while Greenhouse–Geisser was used for not normally distributed data. A two-sided *p* value of <0.05 was considered statistically significant. Statistical Package for Social Science version 23 was used for performing the statistical analysis (SPSS Inc. Released 2015. IBM SPSS statistics for windows, version 23.0, Armnok, NY:IBM Corp.).

## 3. Results

A total of 103 patients with HCV GT-4 infection who failed to achieve SVR12 after SOF + DAC ± RBV were treated per protocol with SIM + SOF + DAC + RBV for 12 weeks. The mean age was 54.48 ± 4.81 years, and 56 (54.4%) patients were male. Baseline demographic and clinical characteristics are shown in (Table 1).

There were no significant changes in the level of follow-up of white blood cells (*p* value = 0.625), platelet (*p* value = 0.580), serum creatinine (*p* value = 0.43), and total bilirubin when they were compared to the pretreatment level or 12 weeks after treatment. Pretreatment hemoglobin was significantly higher than week 4 (0.008) and, then, in all other weeks (*p* < 0.001); also, it was significantly higher at week 4 when compared to its level at week 8 and week 12 (<0.001).

Hemoglobin was significantly higher at week 12 after treatment than its level at week 8 and and week 12 (<0.001). Pretreatment levels of ALT and AST were significantly higher than in all weeks of treatment (*p* < 0.001) (Table 2).

Assessment of the effectiveness of antiviral treatment: end of treatment response was achieved in all 103 patients (100%) at week 12, while SVR was achieved in 100 patients (97.1%) at week 12 after treatment (Table 3).

The safety of antiviral treatment: fifty patients (47.5%) had side effects in the form of anemia in 30 patients (29.1%), pruritus in 43 patients (41.7%), headache in 5 patients (4.9%), fatigue in 22 patients (21.4%), insomnia in 7 patients (7.8%), diarrhea in 6 patients (5.8%), nausea in3 patients (2.9%), cough in 6 patients (8.7%), and myalgia in 2 patients (1.9%) (Table 4).

## 4. Discussion

Our goal in this study was to assess the efficacy, safety, and tolerability of sofosbuvir, daclatasvir, simeprevir, and ribavirin in 12 weeks treatment in direct-acting antiviral-experienced patients. One hundred and three genotype 4-compensated Child-Pugh A cirrhotic patients were enrolled in the study.

Regarding biochemical parameters, basal hemoglobin was significantly higher than its levels in week 4 (0.008) and all other weeks (*p* < 0.001). Moreover, the hemoglobin level at week 4 showed significant increment than its levels at week 8 and week 12 (<0.001). At week 12 after treatment, hemoglobin significantly increased than at w 8 and w 12 (<0.001), which was mainly attributed to the ribavirin effect. Baseline levels of ALT and AST were significantly higher than at all weeks of therapy (*p* < 0.001).

End of treatment response was achieved in all 103 patients (100%), while SVR12 was achieved in 100 patients (97.1%) at week 12 after treatment.

Only the IMPACT study evaluated a ribavirin-free version of the same regimen administered for 12 weeks in 40 treatment-naive or treatment-experienced patients with decompensated cirrhosis or portal hypertension. There was discontinuation of treatment due to adverse events occurred, and a 100% SVR12 rate was achieved. This difference can probably be explained by the fact that our patients had already experienced a failed DAA-based regimen [35].

At the level of real world studies, our findings were similar in regimen but higher in SVR to the results of Hézode et al. [32], where they evaluated the effect of sofosbuvir, daclatasvir, simeprevir, and ribavirin 24-week regimen in 10 direct-acting antiviral-experienced patients. The study included genotypes 1, 2, 4, and 6. Relapse occurred in 2 patients, and they were of genotype 1.

The inclusion of many HCV genotypes in the study of Hézode et al. together with the small number of their patients may explain the difference between the SVR 12 percentages in our study [32].

Our results was quite similar to those of a recently published Egyptian study of Said et al. 2020 who used the same regimen and recorded 96% of SVR12 [36]

Similar studies, but without the same drug regimen, were conducted. In 2015, Lawitz et al. carried out a ribavirin-free regimen (SOF, DAC, and SIM) for 12 weeks in 40 treatment-naive or treatment-experienced patients with decompensated cirrhosis or portal hypertension. There were no side effects, and SVR12 was 100% [35].

Another study aimed to test the efficacy of 12 weeks treatment with SOF plus SIM, without RBV in NS5A-experienced patients. They enrolled 16 patients (genotype 1: 14/16 and genotype 4: 2/16) with advanced fibrosis or compensated cirrhosis. SVR12 was 87.5; the two patients who relapsed were infected with HCV GT 1a, cirrhotic, had relatively high HCV-RNA levels at baseline, and had at least one RAV detected in both the NS3 and NS5A regions [37].

On the same track; Wahsh et al. [38] evaluated the safety and efficacy of simeprevir plus sofosbuvir in 12 week treatment of HCV-naïve and -experienced 175 cirrhotic and noncirrhotic patients, where the liver enzymes showed significant decline at the end of therapy, and SVR12 was achieved in 97.7% of cirrhotic patients who showed lower SVR (92.7%). Mild and tolerable adverse effects were detected in 57.14% of patients mainly in the form of headache, fatigue, pruritus, dizziness, and photosensitivity [38, 39].

As a comparison between our findings and the FDA-approved VOSEVI regimen which is a combination of three antiviral drugs, sofosbuvir (SOF) and velpatasvir (VEL) and voxilaprevir (VOX), this combination was approved to be used in treatment-experienced chronic HCV patients without cirrhosis or with compensated cirrhosis, on the basis of two clinical trials of 748 chronic HCV patients [40].

In our study, the SVR rate was also comparable to those reported by Bourlière et al. in the POLARIS-1study [40], in which the triple combination of SOF/VEL/VOX was used for 12 weeks to treat CHC patients after an unsuccessful NS5A-containing DAA regimen. The overall SVR rate was 96% (253/263) of treated patients, while the SVR was 91% in patients with HCV genotype 4 (20/22 patients) [40, 41].

Regarding the safety of the used antiviral regimen, fifty patients (47.5%) had side effects in the form of anemia in 30 patients (29.1%), pruritus in 43 patients (41.7%) headache in 5 patients (4.9%), fatigue in 22 patients (21.4%), insomnia in 7 patients (7.8%), diarrhea in 6 patients (5.8%), nausea in3 patients (2.9%), cough in 6 patients (8.7%), and myalgia in 2 patients (1.9%).

The relative high percentage of anemia is attributed to ribavirin in the used regimen and to cirrhosis of the study population.

Simeprevir is known to induce many skin adverse effects, the commonest of which is severe itching that explains the obvious pruritus rate in our patients [39].

On the other hand, Hézode et al. [32] reported that 2 patients met severe side effects such as pulmonary arterial hypertension and acute-on-chronic liver failure which may be attributed to the presence of cirrhosis and NS5A and/or NS3 protease resistance-associated [22, 33, 34].

The main limitation of the study is the limited sample size. So, more studies on the broader number of patients with genotype 4 are required to document the safety and efficacy of this regimen on this special category of patients.

In conclusion, the combination of sofosbuvir (400 mg), daclatasvir (60 mg), and simeprevir (150 mg) with a weight-based ribavirin dosage (1000–1200 mg/d) is effective with moderate tolerable side effects in retreatment of compensated cirrhotic patients who failed to respond to previous DAA-containing regimens.

## Figures and Tables

**Table 1:** Baseline demographic and clinical characteristics (*n* = 103).

Variable	Pretreatment mean ± SD, range
Age	54.48 ± 4.81, 27.0–71.0
Sex	
Male	56 (54.4)
Female	47 (45.6)
Hypertension	34 (33.0)
DM	31 (30.01)
FBG	103.72 ± 43.32, 71.0–299.0
HbA1c (*n* = 27)	7.62 ± 0.66, 6.60–8.10
AFP	13.87 ± 38.57, 0.70–276.0
HBs-Ag	
Positive	0 (0.0)
Negative	103 (100.0)
Prothrombin activity	80.66 ± 10.54, 55.0–100.0
Serum albumin	3.79 ± 0.47, 3.0–4.80
Viral load	414722.16 ± 766382.13, 654.0–4002092.0
End of treatment response	100 (100.0)
Sustained viral response	
Positive	3 (3.0)
Negative	97 (97.0)
Ribavirin modification	
Positive	8 (8.0)
Negative	92 (92.0)
Previous treatment	
Sof + DAC	58 (58.0)
SOF + DAC + RIB	42 (42.0)

**Table 2:** Baseline and follow-up laboratory investigations of the studied group (*n* = 103).

Variable:	Pretreatment mean ± SD	W4 mean ± SD	W8 mean ± SD	W12	W12 post	*p* value
Serum creatinine	0.89 ± 0.25	0.89 ± 0.23	0.93 ± 0.26	0.90 ± 0.14	0.90 ± 0.11	0.43
WBCs	6613.0 ± 2298.8	6756.00 ± 2244.51	6721.0 ± 2046.3	6626.5 ± 1909.9	6632.0 ± 1857.3	0.625
HB	13.65 ± 1.70	13.11 ± 1.59	12.16 ± 1.76	12.12 ± 1.88	12.90 ± 1.18	<0.001
Plt	152.51 ± 47.69	160.78 ± 65.09	154.10 ± 56.17	163.07 ± 55.88	161.25 ± 52.04	0.580
ALT	53.39 ± 35.28	27.71 ± 13.28	24.77 ± 10.60	24.85 ± 6.87	24.59 ± 5.89	<0.001
AST	51.55 ± 37.35	28.83 ± 14.28	25.24 ± 9.36	26.07 ± 6.18	25.82 ± 5.38	<0.001
Total bilirubin	1.10 ± 0.45	1.24 ± 0.70	1.16 ± 0.61	1.21 ± 0.51	1.13 ± 0.37	0.104

**Table 3:** Virological response of studied patients.

Time	SVR	95% CI
End of treatment response	103/103 (100.0%)	100.0–100.0
Sustained virological response (12 weeks after treatment)	100/103 (97.1%)	93.2–100.0

**Table 4:** Side effects in the studied patients.

Side effects	No. (%) (*n* = 103)
^*∗*^No side effects	53 (51.5)
^*∗*^With side effects	50 (47.5)
Anemia	
Pruritus	30 (29.1)
Headache	43 (41.7)
Fatigue	5 (4.9)
Insomnia	22 (21.4)
Diarrhea	7 (7.8)
Nausea	6 (5.8)
Cough	3 (2.9)
Myalgia	6 (8.7)
	2 (1.9)

## Data Availability

The authors' institution does not allow public data access.
